# The mental health experiences of ethnic minorities in the UK during the Coronavirus pandemic: A qualitative exploration

**DOI:** 10.3389/fpubh.2022.875198

**Published:** 2022-10-06

**Authors:** Tine Van Bortel, Chiara Lombardo, Lijia Guo, Susan Solomon, Steven Martin, Kate Hughes, Lauren Weeks, David Crepaz-Keay, Shari McDaid, Oliver Chantler, Lucy Thorpe, Alec Morton, Gavin Davidson, Ann John, Antonis A. Kousoulis

**Affiliations:** ^1^Faculty of Health and Life Sciences, Leicester School of Allied Health Sciences, Gateway House, De Montfort University, Leicester, United Kingdom; ^2^Cambridge Public Health, Department of Psychiatry, School of Clinical Medicine, University of Cambridge, Cambridge, United Kingdom; ^3^Mental Health Foundation, London, United Kingdom; ^4^Department of Sociology, School of Humanities and Social Sciences, University of Cambridge, Cambridge, United Kingdom; ^5^Department of Management Science, Strathclyde Business School, University of Strathclyde, Glasgow, United Kingdom; ^6^School of Social Sciences, Education and Social Work, Queen's University Belfast, Belfast, United Kingdom; ^7^Health Data Research UK, Swansea University Medical School, Swansea, United Kingdom

**Keywords:** COVID-19, coronavirus pandemic, inequalities, mental health, ethnic minorities, BAME ethnicity, United Kingdom

## Abstract

**Background:**

Worldwide, the Coronavirus pandemic has had a major impact on people's health, lives, and livelihoods. However, this impact has not been felt equally across various population groups. People from ethnic minority backgrounds in the UK have been more adversely affected by the pandemic, especially in terms of their physical health. Their mental health, on the other hand, has received less attention. This study aimed to explore the mental health experiences of UK adults from ethnic minorities during the Coronavirus pandemic. This work forms part of our wider long-term UK population study “Mental Health in the Pandemic.”

**Methods:**

We conducted an exploratory qualitative study with people from ethnic minority communities across the UK. A series of in-depth interviews were conducted with 15 women, 14 men and 1 non-binary person from ethnic minority backgrounds, aged between 18 and 65 years old (mean age = 40). We utilized purposefully selected maximum variation sampling in order to capture as wide a variety of views, perceptions and experiences as possible. Inclusion criteria: adults (18+) from ethnic minorities across the UK; able to provide full consent to participate; able to participate in a video- or phone-call interview. All interviews took place *via* MS Teams or Zoom. The gathered data were transcribed verbatim and underwent thematic analysis following Braun and Clarke carried out using NVivo 12 software.

**Results:**

The qualitative data analysis yielded seven overarching themes: (1) pandemic-specific mental health and wellbeing experiences; (2) issues relating to the media; (3) coping mechanisms; (4) worries around and attitudes toward vaccination; (5) suggestions for support in moving forward; (6) best and worst experiences during pandemic and lockdowns; (7) biggest areas of change in personal life. Generally, participants' mental health experiences varied with some not being affected by the pandemic in a way related to their ethnicity, some sharing positive experiences and coping strategies (exercising more, spending more time with family, community cohesion), and some expressing negative experiences (eating or drinking more, feeling more isolated, or even racism and abuse, especially toward Asian communities). Concerns were raised around trust issues in relation to the media, the inadequate representation of ethnic minorities, and the spread of fake news especially on social media. Attitudes toward vaccinations varied too, with some people more willing to have the vaccine than others.

**Conclusion:**

This study's findings highlight the diversity in the pandemic mental health experiences of ethnic minorities in the UK and has implications for policy, practice and further research. To enable moving forward beyond the pandemic, our study surfaced the need for culturally appropriate mental health support, financial support (as a key mental health determinant), accurate media representation, and clear communication messaging from the Governments of the UK.

## Introduction

Globally, the Coronavirus pandemic has caused unprecedented challenges to individuals and societies. According to the World Health Organisation's Coronavirus Dashboard figures as of 29th July 2022, over 572,239,451 million cases of infection and over 6,390,401 million deaths have been reported to date. This global crisis has not only resulted in major physical health and health systems challenges; it also has caused significant economic and social disruptions, which further contribute to mental health deterioration (1–4).

However, the effects of the Coronavirus pandemic are not felt equally throughout the population. This pandemic has both exacerbated existing inequalities in society (with vulnerable and socially disadvantaged groups suffering disproportionately), and has created new ones (5–7). It has also been found that people who identify as being from an ethnic minority population group, have higher risk of infection, have been disproportionately hospitalized and have a higher COVID-19 mortality rate compared to people from a white ethnicity in the UK, USA, and elsewhere (8–12). Factors linked to this higher mortality rate include poor socio-economic status, perilous employment, genetics, chronic diseases, co-morbid medical conditions, housing, mental illness, racial discrimination stress, and other social disadvantages (13–17).

This differential impact is currently a particular emerging public health challenge in the UK (18, 19) which could be, in part, due to certain UK sectors' (including health and social care) heavy reliance on people from ethnic minorities [in the UK often referred to as “BAME” (Black, Asian and Minority Ethnic) people] who make up a significant amount of its workforce, alongside various other socio-economic inequalities. These intersecting factors of “ethnic minority” and “lower socio-economic status” mean that people with this particular background have higher levels of poverty in their communities compared to the white population, live often in overcrowded households (20), are less aware of the need for personal protective equipment (PPE) (21), and are more likely to experience job loss and not receive furlough (UK government financial support) compared with UK-born white British people during the Coronavirus pandemic lockdowns (22).

The effects of the pandemic on mental health are also unequal (23). Since the start of the pandemic, researchers have been investigating its effects on mental health among different population groups and have been noting the unequal impact on people from ethnic minorities (24–26). Research highlights that people from ethnic minority communities may be more likely to experience higher levels of distress, anxiety, stigma, and racism during the pandemic (8, 27), and levels of self-harm, abuse and thoughts of suicide and self-harm are higher (28). For healthcare workers from ethnic minority backgrounds in the UK, for instance, more than 70% were anxious about their role during the Coronavirus pandemic (18). This emerging evidence highlights that people from ethnic minority communities are experiencing adverse mental and physical health impacts as a result of the Coronavirus pandemic, thus indicating the importance of ensuring that any mental and physical health responses must address the divergent needs of people from ethnic minority communities.

Gibson et al. (29) have conducted a systematic review of 117 studies that examines the impact of inequality factors on mental health during the Coronavirus pandemic, including “education, income, employment, occupation, material and social deprivation, age, immigrant status, sexual orientation, functional health, cultural/racial background, sex, gender, and place of residence.” Among these studies, only ten studies examined the effect of minority ethnicity and migrant status on mental health, most of which found a negative impact, while some research found no significant difference for race or ethnicity (30). Thus, more in-depth and nuanced research is still needed to understand how ethnicity and intersectionality of ethnicity and other inequality factors may influence the mental health of these population groups in the long run. Researchers are calling for more actions to conduct inclusive research to tackle the challenges of inequality issues, so as to prepare the public health and mental health systems and policy to better support population mental health and cope with further outbreaks (31–33).

Therefore, the aim of this qualitative research was to better understand the pandemic mental health experiences of UK adults who identify as being from ethnic minorities. We have done this through conducting in-depth interviews with people from this population group. The findings from the present study could provide recommendations for policy and practice to ensure that the mental health needs of people from ethnic minorities in the UK are properly understood and accounted for as we move forward through, and out of, the pandemic.

### Research questions

What are the pandemic mental health experiences of UK adults from ethnic minority backgrounds?

What are their further pandemic experiences and coping strategies as well as their views regarding moving forward through, and out of, the pandemic?

### Study aims

The overall aim and objectives of this qualitative research were to better understand the impact of the pandemic and related measures on the mental health of UK adults from ethnic minority backgrounds and how they coped, as well as to explore any potential differences they may perceive in experiences compared to people from other backgrounds. Additionally, we aimed to identify implications for policy, practice and further research to ensure that the mental health needs of people from ethnic minority backgrounds in the UK are properly understood and accounted for.

## Materials and methods

### Study design and setting

This qualitative research utilized a thematic analysis method allowing us to identify, describe and understand the mental health experiences of UK adults from ethnic minorities during the Coronavirus pandemic as well as detecting repeated patterns that emerged across the data and interpret these in context (34).

The qualitative research presented here derives from semi-structured interviews around the topic “mental health experiences of people from ethnic minorities in the UK during the Coronavirus pandemic,” which forms part of our UK-wide “Mental Health in the Pandemic” study, a long-term repeated cross-sectional mixed-method investigation with an embedded qualitative work stream. In this broader study, UK-wide representative cross-sectional surveys were collected, with qualitative research (individual and group interviews) tailored around the emerging survey findings, combined with relevant emerging literature and media reporting in relation to the mental health impacts of the Coronavirus pandemic in the UK. Further details can be found in our published Study Protocol (35).

### Recruitment

Following the repeated cross-sectional survey findings, we conducted 30 in-depth interviews on topics of importance and concern in relation to the “mental health experiences of people from ethnic minorities in the UK during the Coronavirus pandemic.” We utilized purposefully selected maximum variation sampling in order to capture as wide a variety of views, perceptions and experiences as possible. Potential participants were approached through a qualitative research recruitment company, with a specific assignment to recruit a maximum variation sample of participants from ethnic minority communities across the UK.

Inclusion criteria: adults (18+) from ethnic minorities across the UK; able to provide full consent to participate; able to participate in a video- or phone-call interview.

Potential participants received an “Invitation Email” with study background information and the interview topic. They could then contact a designated study person if they wished more information or clarification regarding the study. When a potential participant decided to take part in the study, they contacted the designated study person who provided the participant with a written Consent Form prior to any virtual meetings. Participants were given at least 24 h to decide whether or not they wanted to take part in the study. Those who did take part received reimbursement for their time. Participants were also reassured about the confidential nature of the study and that they could withdraw consent at any time.

### Data collection procedure

The interviews were carried out entirely virtually *via* Zoom or MS Teams, and lasted for ~1 h. The interviews were conducted by expert researchers from the UK-wide “Mental Health in the Pandemic” study consortium.

At the beginning of the interviews, the researchers began by introducing themselves, giving a brief description of the goals and process of the research. The interview topic guide with semi-structured questions was used to continue the interview.

Interviews were video-recorded upon consent. The interviews were transcribed using an authorized qualitative research transcription service. The interviewers (CL, SS, SMD, AJ, DCK, KH, LK, SM) were all experts in the field of mental health, and all trained in safeguarding procedures in research, and data protection for field notes and data collection.

Our interviews were guided and framed by the evidence generated by our own repeated UK national surveys as well as from themes emerging from scientific evidence.

### Data analysis

The data underwent thematic analysis following Braun and Clarke (34), transcripts were read and re-read and line-by-line coding was carried out using NVivo 12 software by CL, KH, LW. Each code's data was checked for consistency of interpretation and further discussed with senior qualitative experts TVB and SS and re-coded as necessary. Further confirmation of themes and contextualization took place through discussions with the wider research team and subsequently with a UK third sector organization tackling racial inequality to improve the lives of ethnic minority communities in the UK.

The researchers who conducted the analysis were experienced in the field of public mental health and qualitative research, with different degrees of expertise in the field of health inequalities. They all came from different ethnic backgrounds [White British, White European, Mixed Background (British-Asian, British-Caribbean)] which brought different views, discussions, contextualization, and understanding during the life of the project.

The semi-structured topic guide research questions were used as an overall framework to develop higher-order themes, together with the interview notes and a preliminary scan of the transcripts. Final confirmation of themes took place through team discussions with the qualitative leads CL and TVB.

The data are presented further below in the form of a summary of key themes evidenced with illustrative quotes.

### Ethics approvals

Ethics approvals were obtained from the University of Cambridge Psychology Research Ethics Committee (No. PRE 2020.050) and from De Montfort University Faculty of Health and Life Sciences Research Ethics Committee (No. REF 422991).

## Results

### Demographic information

We conducted 30 semi-structured interviews with 15 women, 14 men, and 1 non-binary person from ethnic minority communities across the United Kingdom, aged between 18 and 65 years old (mean age = 40). Demographic information on ethnic origin, gender, marital status, age, and occupation is shown below in the Tables 1–4. A few respondents were not comfortable with providing certain demographic information which has been categorized as “prefer not to say.”

**Table 1:** Demographic information on ethnic origins (*n* = 30).

**Ethnic origins**	**Frequency**	**Specific ethnic origin**	**Frequency**
British Asian	11	Bangladeshi	3
		Chinese	2
		Indian first generation	1
		Indian second generation	2
		Pakistani	3
British Black/African/Caribbean	9	African	3
		African first generation	2
		Caribbean	4
Multiple ethnic groups	5	White and Asian	1
		White and Black African	2
		White and Black Caribbean	2
Other ethnic groups	1	Arab (Egyptian)	1
Prefer not to say	4		4

**Table 2:** Demographic information divided by gender and marital status (*n* = 30).

**Marital Status**	**Gender (frequency)**		
	**Females**	**Males**	**Non-binary**
Single	6	7	
Married	6	6	
Divorced	1		
Co-habiting	1	1	1
Prefer not to say	1		

**Table 3:** Demographic information divided by gender and age (*n* = 30).

**Age group**	**Gender (frequency)**		
	**Females**	**Males**	**Non-binary**
18–24	4	2	
25–44	6	7	1
45–65	5	5	

**Table 4:** Demographic information on occupations (*n* = 30).

**Occupations**	**Frequency**
Administrator	1
Ambulance driver	1
Assistant manager	1
Catering assistant	1
Civil service worker	1
Concierge	1
Cyber security consultant	1
Early retirement	1
Executive search	1
Foster parent	1
Freelance photographer	1
IT manager/private tutor	1
Live-in care worker	1
Online retailer	1
Police community support officer	1
Process engineer	1
Recruitment consultant	1
Sales advisor	1
Self-employed (street food industry)	1
Starting own business	1
Students	4
Unemployed	2
Work coach	1
Prefer not to say	3

### Themes and sub-themes

The seven main themes emerging from the interviews focus around (1) the respondents' pandemic-specific mental health and wellbeing experiences, (2) issues relating to the media, (3) coping mechanisms, (4) worries around and attitudes toward vaccinations, (5) suggestions for support needs in moving forward, (6) the best and worst aspects of the pandemic and lockdowns, (7) the biggest areas of change in their lives. Table 5 below is a summary of the different main themes and related sub-themes that emerged.

**Table 5:** Main themes and related sub-themes.

1. Pandemic-Specific mental health and wellbeing experiences	a) Being integrated and sense of identity
	b) Mental health
	c) Physical health
	d) Public reactions: racism and abuse
	e) Determinants of COVID-19
2. Issues relating to the media	a) Lack of trust in the media
	b) Inadequate representation of ethnic minority communities
	c) Fake news
3. Coping mechanisms	a) Positive coping mechanisms
	b) Negative coping mechanisms
4. Worries about and attitudes toward vaccinations	a) Vaccine concerns and hesitancy
	b) Vaccine positivity
5. Suggestions for support in moving forward	a) Mental health support
	b) Trust
	c) Financial and community support
	d) Clear government communications
6. Best and worst experiences during the pandemic and lockdowns	
7. Biggest areas of change in personal life	

#### Pandemic-specific mental health and wellbeing experiences

##### Being integrated and a sense of identity

In general, respondents described themselves as being well-integrated in the cities and towns where they live, referring to their wider communities as multicultural. Providing a positive example from the NHS, one respondent—who described themselves as British Asian—felt that ethnic minorities have not been treated unfairly because of their background:

“*We have a great National Health Service, you know, everyone is welcome, everyone's looked after. It shouldn't matter whether you're Black, White, Asian, whatever. And I don't think it does, not from what I've seen*.” (28M63SM)

Another respondent commented how both the events in America (e.g., the death of George Floyd and the Black Lives Matter protests) and the news that ethnic minorities could face a heightened risk of contracting COVID-19, enhanced a sense of ethnic identity:

“*I think whenever there's anything specific going on that has a big impact. Especially when all the information was coming out; I've met people from BAME communities who are at high risk of complications from COVID. I think again, that highlights again my ethnicity and everything that was happening in America at the time, George Floyd …. it does highlight for me again, the sense of identifying as a minority group and all the, all the kind of, impacts of that. So, I probably maybe thought more about my ethnicity in the last few months than I have done for maybe the two years previously, something like that, you know*.” (1520LW)

##### Mental health

Themes that came out through the interviews included people feeling lonely and isolated, needing better access to mental health service. The pandemic had been difficult for them with regards to processing the impact of death and the feelings of overall grief that this induces:

“...*The mental health impact of death because I think there is nobody who can acclimatize himself with the impact of death… I have never been exposed to death in the manner that I've been exposed because of this situation…*” (5M56CL)

People spoke of an increased sense of anxiety and a sense of uncertainty, and vulnerability due to ethnic origins, which was then eased by clear information:

“*…because we didn't know what we were dealing with. And then being of African origin, there was so much information coming out, well, saying Africans were really susceptible {to COVID-19}. So there was a bit of anxiety creeping in. I can be… I could say I'm a scientist by background but there was a bit of anxiety coming in because we never did know what we were actually dealing with. So it took a bit of time for us really to sort of run with the thing. And this was only after… because only after information started coming out and we were reading information and then that gave us a sense of security and sort of laid off the anxieties*.” (5M56CL)

There were concerns around family members, or they felt their family members were overanxious. One person was worried about the lack of public health restrictions back in their home country, whilst another respondent felt limited empathy from British people in relation to what was happening in their home country. One person felt very isolated from their own community and support network, as they were living with a partner belonging to a different ethnicity and felt that during lockdown they had lost contact with their community:

“...*it just felt like I was very isolated from my community and support network, and also going through that on top of COVID, I felt a bit overwhelmed…*” (24F24AJ)

The pandemic disrupted the lives of our respondents and caused uncertainty about the future, or as one respondent put it that they “*had nothing to look forward to*.” (22M40RB)

The upheaval that the pandemic caused also meant that usual social networks and activities people used to maintain wellbeing were disrupted.

“*I was lacking a routine and I think that didn't help, you know, I didn't have that release of work or release of football or golf or anything else that I wanted to do…*” (22M40RB)

Some said that they had sought help from a counselor during the pandemic to support their mental health as they recognized that they were struggling. Of particular relevance, was to identify support that would match own cultural background:

“*And I think as well now I do have a therapist who is from the same background as me, it's really nice, because I'm explaining things to her and I don't have… I'm talking like…I'm telling her about myself and I don't have to explain every little bit, she will just understand it because she'll understand the cultural nuances without having to… I don't know, it's hard enough explaining your life without having to explain every single bit because the person you're speaking to has no idea…*” (28M63RD)

Those who were younger, expressed a feeling of grief for the loss of a year and the loss of opportunities and experiences they could not have as a result of the pandemic.

“*I'm like, oh God, wasting our youth just, don't know, baking banana bread…it just feels like you're mourning the life you could have had or would have had if not for the pandemic*.” (24F24AJ)

##### Physical health

Participants spoke of different impacts on their physical health. For some the lockdown had been positive and they felt as though they were doing more exercise as there was more time to be able to engage in physical activity, such as:

“... *going for bike rides and walking …*” (2M29KH)

A number of participants engaged with new physical hobbies and engaged in physical activity with their children. They identified wanting to continue with these new hobbies as they not only felt the positive impact on their physical health but also on their mental health:

“*It does interlink with your mental health, as well as your physical wellbeing…*” (29M18DCK)

Others linked the pandemic with increased eating and one participants spoke of the potential impact that could have on them managing their type 2 diabetes. A few corroborated the feeling that the pandemic had a negative impact on their health and spoke of the difficulties of having a workplace and home in the same space and the negative consequence that this had on physical activity:

“... *Not done any movement but again it's hard when your office and your home is in the same place*.” (9M30SM)

##### Public reactions: Racism and abuse

Some respondents reported indirect experiences of verbal abuse and racism. One respondent, originally from Hong Kong, had previously experienced SARS when living there, so they were already prepared to follow the public health restrictions in place. They perceived that some ethnic minorities were treated unfairly due to their background. One other respondent noted an increased avoidance of primarily people who appeared to have a Chinese background as well as other ethnic groups:

“*From around late January, you know, February times, some people were avoiding sitting next to Chinese people or anyone they see to be Chinese. And then, I started to notice, it wasn't just happening, you know, to Chinese people, people were then avoiding sitting next to Black people or South-Asian people, or anyone who didn't look European. In my analysis, and I say this because I've lived here through the 70s and 80s where racism, oh, god, it was terrible*.” (10M36DCK)

Public reactions in some cases were limited to staying away or people staring. One respondent with Asian-looking features, described their feelings and reactions in those situations:

“*Sometimes people tend to stare a bit more often, especially because we're not required to wear the masks when we're just out in the open air, public spaces. I feel like they would look at me a bit funny. Not all the time and not everyone of course, but you do get these nasty glances. […] I'm guessing it's because they think I'm from China, which I'm not, I've never been to China before in my life. For a moment it would make me feel a bit small, but knowing that I have my rights in this country, I was born here, so it didn't…like I say, it didn't affect me too much.”* (16F20SS)

In a more extreme example, one respondent reported experiences of physical violence toward East-Asian men:

“*… made us realise that there is a lot of injustice and segregation, racially, within society*.

*There were a couple of East-Asian men that were beaten up in London because of it {the pandemic}*.” (18F33SS)

One person also noted how racist comments were more common on social media, rather than in person:

“*I think, being on social media, I think, people are a lot more bold to say things that they'd never say in person, because they can hide behind a screen. And so, that was frustrating, I think, dealing with that, {…} in the UK racism is systematic, it's through more verbal actions, it's through suggestion,* it's through phrases…” (7F22KH)

We also noted that some respondents from African background reported mental health impacts because of Mr George Floyd's death in the USA on 25th May 2020 and the subsequent global Black Lives Matter protests that followed. These events also raised further awareness of more systemic racism in the UK and were experienced by many as very upsetting. Whilst these events did not directly relate to the Coronavirus pandemic, they happened during the initial stage of the global pandemic, affecting public protests (especially whilst lockdown restrictions were in place), and compounding pandemic stresses.

##### Determinants of COVID-19

Respondents identified a number of possible reasons for the high prevalence of COVID-19 in ethnic minority communities. Some suggested there could be genetic reasons, while others highlighted social and cultural factors:

“*Some people have a bigger family dynamic which means that they're more likely {to be infected} and because there are more people, as we know, if somebody's unwell, the whole family's going to be here… And socioeconomic, because if people aren't being paid well, sometimes they don't eat well so they're going to be more likely {to fall ill} … and that's in general, and it seems to me, it evades dealing with what has been a problem for probably about 30 years is people not being paid properly and being able to live a comfortable {life} … not an excessive, but a comfortable life with the necessities…*” (21M49LW)

A couple of respondents from British-Asian background highlighted the fact that within their communities, family groups are often larger and more likely to interact in larger groups, with one respondent highlighting that there was a sense of “obligation” to attend important life events such as funerals where people meet in large households and had larger bubbles because of the strong cultural values inside their communities:

“*Because the way that society is structured, you're more closely-knit, the chances of community transmission are very high. If there are weddings and there are any religious things, and also funerals and stuff, I know that… It's like because you're from the community, it is expected of you to be present*.” (12F47KH)

“*You're expected to show that you're in solidarity with the person. I don't necessarily think it is the most practical thing to do, especially in the middle of a pandemic, but I think emotions overpower your reasoning at that time. No, this is what I want and this is how it should be, and you don't necessarily make the best choices or decisions*.” (12F47KH)

However, it was also reported that the majority of respondents from those communities had closely adhered to the physical distancing rules and had taken them very seriously.

*“...I see, like, a lot of ethnic minority families, like, they are way more strict a part so when there are rules being in place, they would tend to be more strict with the rules…*” (29M18DCK)

#### Issues relating to the media

##### Lack of trust in the media

Most respondents felt frustrated by the negative and pessimistic narratives presented by the media during the pandemic. Some concerns were raised with regards to the mainstream media having a hidden agenda when reporting the news and that for many respondents this had resulted in an overall lack of trust in the news:

“*I have a longstanding distrust of the British media and I'm not sure it's because I'm Black because I know for sure that all the people, Asian and Whites, have the same mistrust for them because they all seem to be pursuing an agenda and they seem to walk hand in hand with the government. So that does not help and that has been magnified in this period of pandemic. They are not working in the interest of the general populace, it's quite clear.”* (30F30SM)

For some, the media played a key role in portraying some communities as being less careful when it came to following public health guidance, thus causing resentment in others. For example, one respondent reported:

“*I have felt they've maybe been treated … or viewed upon or looked upon slightly unfavorably by some, and not seen as being as sensible as other communities. I mean, a poor example is probably if I use… I'll use the local area to us, so there's an area called…[omitted]...near where I used to live. And… excuse me, but… it's a very heavy Indian community. And there was loads in the press around how they just didn't care for the virus, how they were seeing their friends and family and it wasn't important to them and they were the ones being the spreaders…*” (1M28SS)

It was also suggested that the portrayal of ethnic minority communities as “virus spreaders,” had an impact at different levels including that some parts of the wider public blamed ethnic minorities for the high infection rates:

“*And I think that was a really unfair attitude and a very easy target because people bought into it and I think the further right-wing some of the newspapers go at the moment anyway made it easy for them to be a target. And people bought into that story. And people began to believe it and blame those from an ethnic minority. Not because they had different skin colors or [were] from a different background but because it was an easy group to attack and target and blame somebody but yourselves.”* (23F24SS)

##### Inadequate representation of ethnic minority communities

For others, there was a lack of representation of different ethnic minorities, with the media paying more attention to some rather than others. Such unequal attention within the media meant that some respondents identified that their experience of the pandemic, and that of their community, had failed to be recognized within the public sphere. For example, a respondent said:

“*I think the media tends to focus more on the Asian side where they are more, I guess, in a way Indian or Pakistani, I guess. I don't want to be racist or anything, but I just feel like they {media} focus more on that sort of Asian ethnicity rather than the Chinese. Because hardly have I seen a Chinese person in the media, so I can't really say that the Chinese experience in a way has ever been … if you discount the Wuhan or people actually from China being interviewed or something, but the Chinese living in the UK, I don't think that they've been represented enough in the media*.” (27F63SM)

Other respondents felt that the media had not picked up on, or spoken in depth about, the increased vulnerability of ethnic minority groups. Participants spoke of how they found it frustrating that as data indicated that people from ethnic minority backgrounds were at increased risk of mortality and morbidity from COVID-19, the media should have done more to explore this and to promote vaccine uptake within those communities. Several participants highlighted that more should have been done to give all higher risk groups vaccination priority:

“*There's so many things being like, oh, ethnic minorities are more at risk for this and that reason, but I'm still not more likely to get the vaccine than someone else. So I'm just a bit like, if we are recognized as a high risk category, why is that not factoring into the decision on how to vaccinate people?*” (24F24AJ)

Furthermore, some respondents perceived that there was a general lack of interest from the media in the experience from people of ethnic minority groups:

“*I guess maybe it's the lack of interest in ethnic minority community or from the lack of interest from the general public, whether it doesn't make a good viewership from them {media}, it's difficult to say, all you see on the news is just updates on the number of deaths or what's happening with the vaccine and stuff like that. I know we've got people from minority backgrounds in the government but that's it, that's all I would see, you wouldn't see a news feature on how someone from an ethnic background has dealt with the pandemic or how it's affected them, once in a while*.”(10M36DCK)

“*But you're constantly seeing news of people losing their jobs, which is from any background, which is fine, you see that on the news anyway but no I don't think there's been much focus on minorities and their experiences, depending on where you get your media from, I guess*.” (10M36DCK)

Only a minority of people felt that media reflected the ethnic minorities experience appropriately, as for example one person said:

“*I can see that, due to my ethnicity, like, more people have either passed {away}, or been affected by COVID really dramatically. So, I feel that the media have portrayed it in a good, well, in an okay way*.” (3F54KH)

##### Fake news

There were many concerns over the spread and the impact of fake news on social media, especially in relation to vaccines, which caused frustration in many.

“*It feels like most of these people are hypnotized by the media, it's like they're controlled to believe in everything, you know, what the news says and they believe in that, and they've not been able to think on their own two feet. So yeah, I think forget about, you know, like thinking about fake news and get yourself vaccinated moving forward*.” (12F47KH)

#### Coping mechanisms

There was considerable variation in the experiences of interview respondents. However, the majority identified that despite the challenges of the pandemic, they had coped well overall.

“*To be honest, we've been fine, I think. We have our ups and downs, but generally we are fine, you know?*” (19M40LW)

##### Positive coping mechanisms

The most common responses were engaging in some form of exercise—walking, cycling, home workouts—and keeping in touch with friends and family:

“*I went for a walk, like one hour, one hour and a half, outside, just to get some fresh air, and I think that helps in helping the mental health…*” (30F30SM)

Respondents emphasized that having support systems from different networks of life—friends, family, work, neighbors, religious community—was useful in meeting emotional and practical needs during lockdown.

“*I think I've coped fairly well, and I think that's because of the sort of support systems that's in place. So as well as having like friends and family at work, the organization that I work for, they put a lot of resources in place for staff…*” (3F54KH)

Religion was also identified as being an important coping mechanism by nearly half of respondents. Solace was gained from both individual and collective worship, with respondents highlighting the importance of being able to attend religious services online throughout lockdown but also seek spiritual comfort on their own at home when they were struggling, for example reading the Koran or chanting:

“*If I'm feeling really low or whatever, the way I was brought up, I will go and read my Bible, I will go and read it*.” (27F63SM)

“*Personally, well, what keeps me going is because of my personal faith…that's where I draw most of my strength... Yes, I have been affected. But I have a support system that is really dependable and that's the word of faith*.”(8M56CL)

A minority of respondents had lost friends or family members during the pandemic—one respondent highlighted how their faith had played an important part in coping with grief and as having provided some comfort:

*“I mean, it's a good thing that I have a firm belief in God, you know, and I believe that He knows best kind of thing, so it's, kind of, with my inner faith… gives me a bit more peace*.” (13NB36KH)

Other coping mechanisms with a positive impact on wellbeing included limiting the consumption of news to avoid over-saturation of negative information and using the relative seclusion lockdown offered as an opportunity to “*catch up on the things*.” (18F33SS)

##### Negative coping mechanisms

During the interview discussions about respondents' coping mechanisms, many people identified that they had eaten more during lockdown. The contrasting boredom and stress of lockdown, loss of routine and altered food consumption habits meant that some respondents reported to eat more than usual or ate more “treat foods:”

“*I've definitely, definitely eaten more than I usually do. It's too convenient. It's too easy. I've got my fridge and my cupboard staring at me whilst I'm working, saying, come and get a snack*.” (1M28SS)

Whilst more than half of respondents said that they did not drink alcohol, a minority said that they had drunk more than usual. One person made the point that for them, this was because of the changes in social interaction during lockdown, with the move to online socializing on Zoom, which had led to them drinking more:

“*In lockdown one, we consumed more alcohol than we've ever done. And I blame the quizzes over Zoom… There was quizzes every other day … and it was just assumed you were drinking…*” (1M28SS)

#### Worries around and attitudes toward vaccinations

##### Vaccine concerns and hesitancy

Several respondents mentioned that they were worried and unsure about COVID-19 vaccinations which caused additional stress. This related either toward worries around potential issues having the vaccine themselves, or loved ones having (or not) the vaccine, or job loss in case of refusing to take the vaccine (e.g., in health and social care professions). Further, respondents who also expressed reservations about the vaccine stated that a lack of trust in the government, historic issues around ethnicity and vaccinations, and the speed at which the vaccine was produced were influencing their decision not to have it. This was identified by several people as creating misgivings in accepting the information being given about the vaccine:

“*I think, over the last nine months, I think, that lack of trust or the broken trust has just deepened over and over and, I think, there's only so many times people are willing to get burnt*.” (7F22KH)

“*I definitely don't believe in the vaccine even being called a vaccine. I think that we as the public have not been appropriately informed. I think we're just getting data that people want us to see*.”(12F47KH)

Additionally, one interview participant highlighted that previous examples of highly unethical medical research on Black people was one of the reasons for skepticism about the safety of the vaccine within their community:

“*Their concerns are based on suspicion, mainly with regards to previous history of how Black people have been exploited for medical reasons and so on, and that's one side of it…*” (28M63RD)

A respondent from a Black African background voiced how belonging to a certain group influenced their thoughts around the vaccine:

“*I am slightly apprehensive about it… I think in my ethnicity, people are a bit more conscious about getting the vaccine…*” (2M29KH)

Concerns were also voiced about the speed at which the vaccine had been developed and the possibility for unknown and unintended long-term side effects. There is a potential correlation between mistrust due to past experiences of racial exploitation in medical testing amongst certain black ethnic minority groups and the perception that the speedy development of the vaccine makes it appear as another *“experiment”* with potentially unknown consequences.

“*For me it's about the speed of the development of the vaccine. Because, you know, normally it takes ten years to develop a vaccine, and I know that they're saying that all the safety measures have been followed… there's no factual information to back that up because people are just having the vaccination now, and you're not really going to be able to see the effects of that until, you know, five, ten years, 15 years down the line…”* (3F54KH)

Several respondents voiced worries and hesitancy about taking the vaccine because they did not know what was in it. For some, concerns about the ethical standards of the vaccine had led to hesitancy about its acceptability on religious grounds. Additionally, the spread of false information, for example, the idea that the vaccine could contain microchips was mentioned by two participants as worrying them and impacting on decision making within their community. The existence of false information was identified by a few respondents as being widespread within ethnic minority groups, with respondents stating that social media had played a big role in spreading rumors and related worries. Misinformation like this can be damaging as it can lead to increased stress, anxiety, hesitancy and resistance to receiving the vaccine:

“*There has been skepticism of the vaccination saying no, it's not halal, some people saying that obviously it's got pork in it. It's like if you get injected, you have a chip inside you, they control you from a chipboard, they control you from a monitor, where you're going, what you're doing and stuff like that…”* (10M36DCK)

“*…like information failure within, especially like my ethnic community and like other ethnic minorities, there's a lot of uncertainty about the vaccine. And they think that, you know, it's like the Government, and, you know, the conspiracy theories…*” (29M18DCK)

##### Vaccine positivity

However, many interview respondents were happy to get the vaccine and saw it as the best available option for protection, both for themselves and for others, and as a viable way to return to some level of normality. Trust in the scientific process was also mentioned as being a causal factor in confidence in accepting the vaccine as legitimate:

“*I definitely agree that everyone should have it, because this perhaps would lead to a normality across the whole wide world…*” (30F30SM)

Despite initial hesitancy, validation of the vaccine from a trusted source provided clarity and peace of mind for some and encouraged uptake. Respondents gave examples of the religious authorities and community leaders, as well as trusted family members providing information and reassurance about the vaccine. This emphasizes the importance of effectively engaging all communities in the co-design and endorsement of information messages and ensuring that all concerns about the vaccine are addressed and information effectively disseminated through appropriate sources:

“*From a religious point of view, I'm thinking, well, is it safe, is it not safe. But then I thought when the British Muslim Association in the UK mentioned that say, for example, it is halal and obviously to move forward, we have to get the vaccination done. There's no harm in getting it done, it's like obviously getting a flu jab or like, you know, when you get a flu jab…*” (10M36DCK).

#### Suggestions for support in moving forward

##### Mental health support

Several respondents expressed the need for increased mental health support, such as increased access to counseling and specific support for healthcare workers:

“*I think counseling is a big thing, I think that's going to hit the roof, not just for myself, I'm talking more so, the hospital staff, what they're going through, I can see that going through the roof, them needing support. Just generally, people though, you know, people are losing their jobs. I think mental health is going to be a big thing*.” (19M40LW)

One respondent stated that they needed to seek some professional mental health support and posed questions of what they needed help with in a post-pandemic world:

“*How can I motivate myself? … How can I get myself confidence? … And how can I face the world again?*” (11F56KH)

Some respondents voiced the urgent need for these services to be resourced and accessible, particularly relating to reducing waiting times when services are likely to become inundated with referrals as we move out of the pandemic as currently:

“...*there's a long waiting list to get the help…*” (16M40KH)

Alongside this increased ask for more counseling services, some respondents spoke of how mental health services need to be culturally appropriate and specifically focus on the needs of people who identify as being from an ethnic minority background. It was recognized that prior to the pandemic there were disparities in mental health care with different cultures and ethnic groups, such as:

“...*issues with delivery of mental health, especially to black and ethnic minorities…*” (28M63RD)

This disparity was likely to have worsened because of the pandemic and may have led to adverse impacts:

“*I'm sure we will have an increased rate of suicide and things like that among ethnic minorities. The damage and the losses and the costs related to COVID are easily recognizable*.” (28M63RD)

Participants also spoke of the need for better cultural competency, a range of accessible therapies in different languages and another spoke of the charity Black Minds Matter who offered them free therapy:

“...*be matched with a therapist who was also from a similar background. So, I've started that during lockdown and it's been good*.” (24F24AJ)

##### Trust

Respondents spoke of the need for rebuilding trust with ethnic minority communities. One participant spoke of how communities needed to be more compassionate:

“...*able to understand one another and just get the help that you need…*” (10M36DCK)

Furthermore, the cyclical relationship of not trusting the government and feeling as they were not trusted that they felt was damaging for mental health and wellbeing:

“...*how are we going to gain the trust of people from different ethnicities and vice versa… Because if it's leading to so much suicides and things like that, you know, there has to be a change…*” (10M36DCK)

##### Financial and community support

There was a mixture of responses regarding the impact on personal finances. Some spoke of how lockdown had helped financially and they have been able to save, for example through working from home and reducing commuting: “...*most money I've ever had…*” (7F22KH)

Others, who described themselves as second generation immigrants praised the current welfare system compared to the one back in their “*home country*” [...] *and felt very lucky to, you know, be living in a country where we can afford to sit at home and keep everyone safe There's countries around the world like, I don't know, people in India that have nowhere to go, so the infection rate there is ridiculous”* (22M40RB).

Some participants discussed how the lockdown had not been as financially rewarding for them and how it had a big negative impact. For instance, people losing jobs or the furlough funding (UK Government support funding during pandemic lockdowns) was less than their usual salary.

For others it lead to increased pressure on making payments such as having to pay for more fuel when driving where they'd usually get free travel on public transport and bills such as mortgage payments:

“....*we have a mortgage and we have had to have ongoing discussion with the lender in terms of reduced payments and it gets tougher each time because it's not the kind of discussions you really want to be having because it's stressful*” (28M63RD).

Participants also spoke of the need for increased funding for struggling charities as there are for services and increased financial support for people, including financial support for students:

“...*if we had a cash payment or transfer or whatever that would help for students going back. Especially with students that had paid tons of their loans to rent, it's affected them*” (15M20LW).

Community support was highlighted as an important theme and participants stressed that support would be needed so that different levels of community support would be able to continue to thrive. One participant spoke of how community support was two-way and that communities need to:

“...*come out in the open so that we can have access to whatever provisions that they are*” (5M56CL).

##### Clear government communications

Finally, participants spoke of how there needed to be clearer messaging from the government around the lifting of restrictions as currently people found this confusing, particularly with the different lifting of restrictions and different rules across the four UK nations (England, Wales, Scotland, and Northern Ireland):

“...*different things happening up in Scotland to England and different things happening in England to Wales and it's just mixed messaging, it's confusing*” (9M30SM).

#### Best and worst experiences during the pandemic and lockdowns

We asked our respondents to reflect on their best and worst experiences of the pandemic and associated lockdowns. Answers were consistent among respondents and can be grouped in the following categories, as set out in Table 6.

**Table 6:** Best and worst experiences during the pandemic and associated lockdowns.

**Best experiences during the pandemic and associated lockdowns**
• Having more time to learn new things and try a different career • Spending more time with family, and at home in general, and with the online social community • Improving own diet and having more time to exercise • Working from home and not having to commute (with one person also saying that the UK Government financial support scheme (furlough) had a positive impact) • Having the opportunity to declutter the house and the garden • Having more time to spend on leisure activities • Enjoying traveling on empty streets and public transports
**Worst experiences during pandemic and associated lockdowns**
• Lack of wider social network (including not seeing family, friends, and work colleagues) • Job uncertainty/employment insecurity • Impact of COVID-19 on family members • Death of family and friends • Missing social activities (e.g., going to restaurants, pubs, and cinemas) • Negative feelings associated with COVID-19 • Government attitude • Some members of the public not complying with public health measures

#### Biggest areas of change in personal life

We asked respondents what the biggest areas of change were for them over the pandemic and lockdown periods. We have grouped their responses around six main areas, as set out in Table 7.

**Table 7:** Main areas of change according to respondents.

**New activities**
Some people started new activities such as, for example, baking and cooking, and doing physical exercise at home
**Employment**
• Some respondents had a career change, either because they lost their job during the pandemic or to accommodate family needs, or due to redundancy • Working from home was the biggest change for many; for many this was beneficial, while one respondent found it frustrating having to go to work and finding that some people were not adhering to the COVID-19 public health measures
**Mental health**
Most respondents found it challenging to be confined to their house, and found it particularly tough not to meet family and friends. However, a few said that they used the time in solitude to reflect more and develop more mental health awareness. One respondent mentioned that they enjoyed the lockdown period
**Physical health**
There were mixed views on the experience of physical health. Some took the opportunity of lockdown to take control of their own diets and to exercise more, whilst others ate more or described their health as going up and down
**Social interactions**
For all respondents, this was the biggest area of change. It included not being able to see friends and family regularly, going to the cinema, pubs and restaurants, or playing sports. Respondents missed not being able to meet the extended family. Some people started online dating, and others took care of family members they were living with
**Travel**
A few people mentioned that they missed traveling to go on holiday, and others were affected by not being able to travel within the country due to the restrictions as they had no family or friends nearby

## Discussion

The current study aimed to gain a better understanding of the mental health experiences of UK adults from ethnic minority backgrounds during the Coronavirus pandemic and its associated measures.

Parkin et al. (36) have reviewed the wider UK policy context in this respect. They highlighted that in February 2022 the Government produced its *Leveling up the United Kingdom* White Paper (37) which includes specific goals to reduce inequities in health, including mental health. NHS England and NHS Improvement's *Advancing Mental Health Equalities Strategy* (38) also acknowledged the importance of addressing inequalities, and the ongoing differences in access, experience and support. It is also the case that these avoidable inequalities in health have been repeatedly identified, including in Government commissioned reports (39–41). The recent mental health policies in Scotland (42), Wales (43) and Northern Ireland (44) all highlight the importance of addressing inequalities in order to promote mental health. In their review of mental health policies in England from 1999 to 2020, Hussain et al. (45) report that over this period, ethnic mental health inequalities remain comparable and policy recommendations have also remained largely the same.

In general, our study findings revealed that the respondents had quite a diverse range of experiences and are sending a strong message of how the experiences of ethnic minority communities are not homogenous, with those who have been in the UK for longer or were born in this country feeling more integrated, regardless of their ethnical background. Hence, relevant research, policies and practices need to be nuanced and avoid generalizations.

Other key differences have been identified in the demographic data from the respondents. The majority of our respondents were in relatively secure jobs, with only two being unemployed and four being students. Various people had the opportunity to work from home which, for most, had a beneficial effect on their quality of life as they could spend more time with family and have a better work-life balance. Nevertheless, there is also evidence that people from ethnic minorities are disproportionately represented in high-risk occupations not able to work from home or, if working from home, not in adequate housing situations contributing toward increased stress and mental health pressures (46). This underlines the importance of addressing the structural inequalities experienced by many people from ethnic minority communities, and having the mechanisms in place to identify and address these. One such example is mandatory ethnicity pay gap reporting, similar to that required for gender. This has yet to be introduced by the UK government, but has been identified by the Women and Equalities Committee as an important indicator employers can use for gaining insights into ethnic disparities that might exist in their workforce so that these can be addressed (47). They cite Equality and Human Rights Commission research, which found that the pay gaps experienced by people from ethnic minorities are largely a result of the challenges they experience with entering employment and progressing at work (48).

Our analysis also raised some of the key benefits and risks of community identity. Some respondents reported that their ethnicity did not affect their experience of the pandemic and described themselves as being well-integrated in their wider, often multicultural, communities in the cities and towns that they live in. However, some respondents had witnessed episodes of racism and abuse, especially toward those from Asian communities. Previous research has also shown that ethnic minorities had experiences of discrimination (49). Black participants in our study reported on their experiences of ethnic discrimination while South-Asian participants reported on their experiences of religious discrimination (50). Participants in our study also discussed around the impact of public health restrictions on ethnic minorities, and the links between COVID-19 cases and communities where large family gatherings are the cultural norm. A study that investigated the impact of COVID-19 and imposed restrictions on Muslim communities in North-West England, for instance, also found negative impacts on psychological wellbeing, livelihoods, and fundamental social interactions of individuals, their families and the wider community. They experienced low mood because their fundamental interactions linked to cultural and religious practices were restricted due to the COVID-19 physical distancing and isolation policies in the UK (51).

This study also found that there was considerable variation in respondents' experiences regarding overall coping during the pandemic. The majority of people identified that, despite the challenges of the pandemic, they had coped well overall. With regard to their behavioral changes and mental health status during the pandemic and lockdowns, some respondents experienced positive changes. Examples of positive coping strategies were changes made for a healthier diet and/or increased exercise levels, staying in touch with family and friends (virtually and, when possible, face-to-face). However, some other respondents also reported negative experiences such as feeling more isolated, and some used negative coping strategies, such as overeating or increased alcohol consumption. There has also been previous evidence suggesting a higher risk of mental health morbidity, feelings of anxiety, loneliness among ethnic minority communities (52–54) and more alcohol use during the initial phase of lockdown (55).

Furthermore, our study respondents raised concerns around media issues, including lack of trust in the media, inadequate representation of ethnic minorities, and the spread of fake news, especially on social media. The issue with transmission of misinformation on social media has also been highlighted in the wider literature (56). Proactive measures should be taken to tackle the rumors and fake news and ensure the effective delivery of accurate, balanced and representative information about the Coronavirus pandemic, COVID-19 disease, and related public health measures, so that people can make informed reasonable judgements and responses. The issue of online misinformation and disinformation is included in the duty of care provisions in the Online Safety Bill currently going through the Westminster Parliament, and the regulatory framework that will follow. It will be important to monitor how effective this is once the Bill is enacted (57).

This study also documented the worries around and attitudes of the participants toward vaccinations. There was considerable variation in responses from interview participants when asked about whether they would, or already have, received a COVID-19 vaccine. Some felt happy and willing to receive a vaccination whilst others were concerned and more hesitant, and some said that they would completely refuse it. The higher vaccine worries and hesitancy amongst people from ethnic minorities has also been evidenced in some previous research. Studies found people with ethnic minority backgrounds and those from socially disadvantaged groups were more likely to reject the vaccine due to common concerns over safety and effectiveness of the vaccine and the perceived lower risk of catching the Coronavirus (SARS-CoV-2) and developing COVID-19 (58–62). Thus, dealing with vaccine hesitancy and social inequalities in vaccine hesitancy among ethnic minorities and other socially disadvantaged groups is crucial to advancing through the pandemic and for future reference. Public messaging should be tailored to address these concerns and use local trusted leaders, as suggested by many health researchers (63–66). This has been recognized by the Cabinet Office Race Disparity Unit, in its final report (67) on progress to address COVID-19 health inequalities. This recommends that Government departments, their agencies and the NHS must build on the local partnerships and networks formed as part of the vaccination programme to continue to build the trust of ethnic minority groups in health services, and that the successful elements of the vaccination programme must also be applied to work to tackle longer-standing health disparities. This has been identified as a priority for the new Office for Health Improvement and Disparities and its partners. The report also recognizes the deficiencies of current data on ethnicity, and recommends improving the quality of ethnicity data coding, and that consideration should be given to how linking of health and Census data could be improved and extended to enable more reliable, timely and detailed estimates of ethnic health disparities on a regular basis.

More generally the theme of “trust” recurred multiple times across our interviews. As effective pandemic response depends critically on trusted institutions, our interview data gives insights into the social fault lines which may undermine an effective social response in both the COVID-19 pandemic and potentially also in future pandemics. Of course it is important that people from ethnic minority backgrounds should see themselves represented in government, media and the public health profession, but our research also shows that religious and community leaders have a critical role in endorsing public health messaging and in ensuring that public health messages are framed in a way which is culturally sensitive and inclusive (e.g., the British Muslim Association endorsing the vaccines as halal).

Additionally, the current study uncovered participants' suggestions and recommendations concerning support in moving forward through the pandemic and beyond. Although a few people did not think any support or other measures were necessary, many participants highlighted how help and support was needed in regard to their mental health (including culturally appropriate mental health support), financial support (as an important determinant of mental health), and that clear pandemic and COVID-19 disease communications from the Governments across the whole of the UK was very necessary in moving forward.

Furthermore, in the UK, the commonly used term “BAME” (Black, Asian, Minority Ethnic) to refer to people from ethnic minority backgrounds is very unhelpful and not correct as it implies that such a group is homogenous and prevents more granular data being presented on individual communities, races, ethnicities and religions in relation to mental health (68). This is also recognized by the Race Disparity Unit's report (67), which recommends that government and health agencies address their work to specific ethnic minority groups rather than a homogenous group, citing use of the term “BAME” as an example of the latter. Achieving this will, however, require focus, discipline, effort and resources, sustained long beyond the experiences of the pandemic, which have shone an especially stark light on the inadequacies of a term that has been relied on for too long.

As suggested by the broad range of risk and protective factors to health that our participants reported, preventing the negative mental health impacts of the pandemic will require a whole-government approach. Measures to address social determinants at the structural level require action by government departments other than health, for example housing, communities and local government, education, justice, transport, and welfare (69).

Although both our current study and previous research have documented mainly the negative impacts of the pandemic, crucial positive changes and habits built through adapting to the reality of the last two-and-a-half years were highlighted in the experiences of our study respondents too. Some participants emphasized the effects and importance of positive coping strategies and that they would continue to maintain these positive changes while moving forward. All these are very important assets to build upon in going forward and to develop new protective factors at individual, community, and societal levels (70).

## Strengths and limitations

The Coronavirus pandemic will likely have a long-term impact on the general population and people from ethnic minority backgrounds in particular. This study has contributed toward presenting a fuller picture of mental health experiences of ethnic minorities, who are still under-represented in research. This study offers recommendations for developing better mental health programmes and policies to support ethnic minority communities, now and in future.

This study was conducted *via* online platforms in order to minimize the risk of Coronavirus infection. However, at the same time, this limited our ability to identify non-verbal cues or contextual factors during the interviews. Furthermore, the online nature of the interviews also meant that the sample is over-represented by certain more advantaged sub-groups within the wider ethnic minority communities, lacked older people and people who are digitally excluded or who do not speak sufficient English to be able to take part in an online interview. Therefore, more disadvantaged and difficult-to-reach sub-groups within the ethnic minority communities were under-represented or not represented. Future research should look into this.

We relied on an external recruiting company to approach our study participants. We acknowledge that for future research more effective engagement strategies would be required to access more-difficult-to-reach participants within their community. It is also worth noting the significant impact that language barriers, stigma and other socio-cultural factors can have on the number of individuals from ethnic minority communities being recruited to health and social care research.

Gatekeepers who support researchers with ethnic minority communities often enable better participation and engagement with a study and its dissemination, and help instill greater confidence in research. Working with local partners who have access to diverse ethnic minority communities would have been also beneficial. Other methods to recruit participants could have included engaging with community, voluntary and faith-based organizations, linking to existing patient groups, giving talks on local, community radio stations, using social media and engaging with relevant community workers (71).

## Conclusions

Overall, our study findings revealed that respondents had quite a diverse range of pandemic mental health experiences, sending a strong message that “people from ethnic minority backgrounds” are not a homogenous group. Those who have been in the UK for longer or were born in this country felt more integrated, regardless of their ethnic background. Further, those from a higher socio-economic status living and working in advantageous circumstances reported to be coping much better than those who were not. Hence, relevant research, policies and practices need to be nuanced and avoid generalizations.

Investigating intersecting factors such as being from a lower socio-economic status, being less integrated, and being from an ethnic minority background is crucial to understand and address the inequalities—including mental health inequalities—experienced by people with this background.

The UK Government, NHS England and the devolved governments of Scotland, Wales, and Northern Ireland all highlight the importance of addressing inequalities in order to promote mental health.

To enable moving forward through and beyond the pandemic, our study also surfaced the need for culturally appropriate mental health support, financial support (as a key mental health determinant), accurate media representation, and clear communication messaging and mental health strategies from the Governments of the UK. Furthermore, it is also clear from this study and our wider pandemic work that the social determinants of mental health as well as leveraging individual and community assets/strengths are crucial in addressing mental health inequalities and promote good mental health for all. This necessitates a “whole government” and “systems” approach, cross-sector partnerships, and meaningful engagement, consultation and co-creative work with all relevant communities.

## Data availability statement

The raw data supporting the conclusions of this article will be made available by the authors upon request.

## Ethics statement

The studies involving human participants were reviewed and approved by Ethics approvals were obtained from the University of Cambridge Psychology Research Ethics Committee (No. PRE 2020.050) and from De Montfort University Faculty of Health and Life Sciences Research Ethics Committee (No. REF 422991). The patients/participants provided their written informed consent to participate in this study.

## Author contributions

TVB and CL wrote the initial manuscript and subsequent iterations and corrections. AK, LG, and SMa helped advance different versions of the manuscript. TVB and AK are joint study leads. AJ, AM, and GD are lead collaborators. SS is the study coordinator. DC-K, SMc, SMa, LG, KH, LW, OC, and LT are researchers on the study. All authors critically reviewed, revised the manuscript for important intellectual content, and read and approved the final version of the manuscript to be published.

## Funding

The study was funded by the Mental Health Foundation UK (Grant No RNAG/635) with matched funding in-kind from De Montfort University Leicester and the University of Cambridge, and additional University-sponsored staff-time input toward the study from Swansea University, Strathclyde University, and Queen's University Belfast, UK.

## Conflict of interest

The authors declare that the research was conducted in the absence of any commercial or financial relationships that could be construed as a potential conflict of interest.

## Publisher's note

All claims expressed in this article are solely those of the authors and do not necessarily represent those of their affiliated organizations, or those of the publisher, the editors and the reviewers. Any product that may be evaluated in this article, or claim that may be made by its manufacturer, is not guaranteed or endorsed by the publisher.
